# Fostering the implementation of liquid biopsy in clinical practice: meeting report 2024 of the European Liquid Biopsy Society (ELBS)

**DOI:** 10.1186/s13046-025-03398-4

**Published:** 2025-05-23

**Authors:** Klaus Pantel, Catherine Alix-Panabières, Paul Hofman, Nikolas H. Stoecklein, Yong-Jie Lu, Evi Lianidou, Patrizio Giacomini, Claudia Koch, Vincent de Jager, Zandra C. Deans, Jennifer Fairley, Simon J. Patton, Rodrigo A. Toledo, Ed Schuuring, Ellen Heitzer, An Hendrix, Franz Lennard Ricklefs, Basant Kumar Thakur, Nikolas von Bubnoff, Jean-Yves Pierga, Christoffer Gebhardt, Claus Lindbjerg Andersen, Remond Fijneman, Núria Malats, Ariane Hallermayr, Claude Chelala, Simon A. Joosse, Gennaro Ciliberto

**Affiliations:** 1 ELBS, Hamburg, Germany; 2 Department of Tumor Biology, University Medical Center Hamburg-Eppendorf, Martinistr. 52, Hamburg, 20246 Germany; 3 Laboratory of Rare Human Circulating Cells (LCCRH) and Liquid Biopsy, University Medical Centre of Montpellier, Montpellier, France; 4 CREEC (CREES), Unité Mixte de Recherches, IRD 224-CNRS 5290-Université de Montpellier, Montpellier, France; 5 IHU RespirERA, Laboratory of Clinical and Experimental Pathology, FHU OncoAge, Biobank 0033-00025, Côte d’Azur University, Nice, France; 6 Department of General, Visceral and Pediatric Surgery, University Hospital and Medical Faculty of Heinrich-Heine University Düsseldorf, Moorenstr. 5, Düsseldorf, 40225 Germany; 7 Centre for Cancer Biomarkers and Biotherapeutics, Barts Cancer Institute, Queen Mary University of London, London, UK; 8 Department of Chemistry, Analysis of Circulating Tumor Cells Lab, Laboratory of Analytical Chemistry, University of Athens, Athens, Greece; 9 Precision Medicine Unit in Senology, Fondazione Policlinico Universitario Agostino Gemelli IRCCS, Largo Agostino Gemelli, 8, Roma, 00168 Italy; 10 Department of Pathology and Medical Biology, University Medical Center Groningen, University of Groningen, Groningen, the Netherlands; 11 Genomics Unit, NHS England, London, UK; 12 GenQA, Nine, 9 Little France Road, Edinburgh BioquarterEdinburgh, EH16 4SA UK; 13 EMQN CIC, Unit 4, Enterprise House, Manchester Science Park, Pencroft Way, Manchester, M15 6SE UK; 14 Biomarkers and Clonal Dynamics Group, Vall d’Hebron Institute of Oncology (VHIO), Vall d’Hebron Barcelona Hospital Campus, Barcelona, 08035 Spain; 15 Centro de Investigación Biomédica en Red de Cáncer (CIBERONC), Institute of Health Carlos III (ISCIII), Madrid, 28029 Spain; 16 Institute of Human Genetics, Diagnostic and Research Center for Molecular BioMedicine, Medical University of Graz, Graz, Austria; 17 Christian Doppler Laboratory for Liquid Biopsies for Early Detection of Cancer, Medical University of Graz, Graz, Austria; 18 Department of Human Structure and Repair, Laboratory of Experimental Cancer Research,, Ghent University, Ghent, Belgium; 19 Cancer Research Institute Ghent, Ghent, Belgium; 20 Department of Neurosurgery, University Medical-Center Hamburg-Eppendorf, Hamburg, Germany; 21 Department of Pediatrics III, University Hospital Essen and University Duisburg, Essen, Germany; 22 University Cancer Center Schleswig-Holstein, University Hospital of Schleswig-Holstein, Campus Lübeck, Lübeck, 23538 Germany; 23 Department of Hematology and Oncology, University Hospital of Schleswig-Holstein, Campus Lübeck, Ratzeburger Allee 160, Lübeck, 23538 Germany; 24 Department of Medical Oncology, Université Paris Cité, Institut Curie, Paris, 75005 France; 25 Fleur Hiege Center for Skin Cancer Research, University Medical Center Hamburg-Eppendorf, Hamburg, Germany; 26 Department of Dermatology and Venereology, University Medical Center Hamburg-Eppendorf, Hamburg, Germany; 27 Department of Molecular Medicine, Aarhus University Hospital, Aarhus, Denmark; 28 Department of Clinical Medicine, Aarhus University, Aarhus, Denmark; 29 Department of Pathology, Netherlands Cancer Institute, Amsterdam, The Netherlands; 30 Genetic and Molecular Epidemiology Group, Spanish National Cancer Research Center (CNIO) and CIBERONC, Madrid, Spain; 31 MGZ – Medizinisch Genetisches Zentrum, Munich, Germany; 32 Scientific Direction, IRCCS Regina Elena National Cancer Institute, Rome, Italy

## Introduction

Despite breakthrough discoveries in biomarker research, implementing them into clinical practice remains a challenge, limiting research benefits for cancer patients. This limitation is also true for the promising field of “liquid biopsy”, which involves analyzing tumor cells or tumor cell products in the blood or other fluids (e.g., urine or cerebrospinal fluid) of cancer patients. The advent of liquid biopsy has opened a new avenue in cancer diagnostics, as highlighted in the latest Nature Milestones Cancer edition in 2020 [1].

**Fig. 1:**
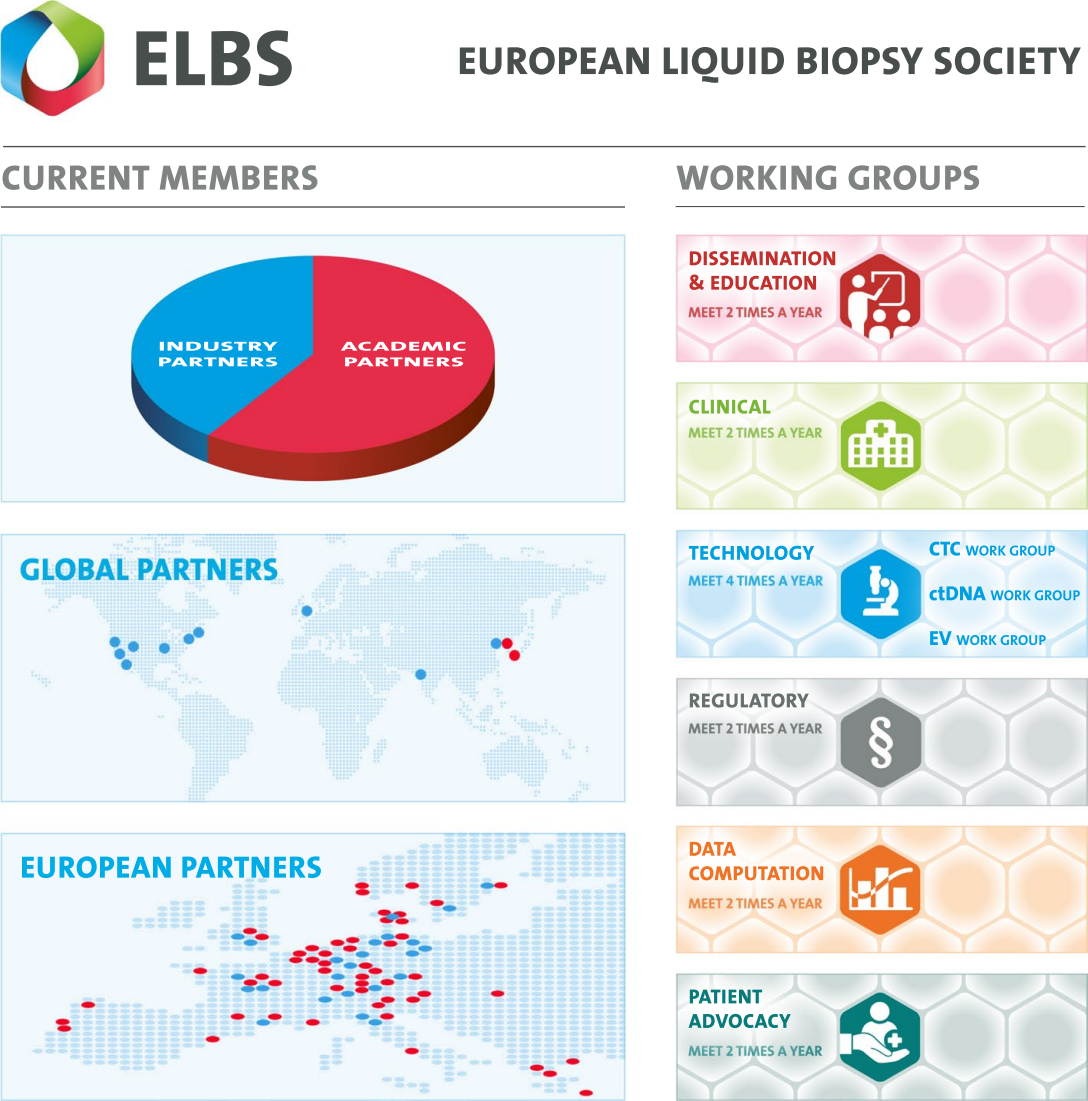
The ELBS, founded by Klaus Pantel in 2020, pursues a holistic approach to tackling the challenges of clinical implementation in the liquid biopsy field. The network incorporates key players from academia, clinic, industry, and national government agencies and currently consists of 93 members from 21 countries world-wide. The ELBS consists of six distinct working groups that are chaired by steering committees of 2–4 members that drive the activity of the network in their specific area of expertise

The ELBS (“European Liquid Biopsy Society”) network is a partnership comprised of member institutions from academia and industry, aimed at advancing liquid biopsy from a dynamic academic field to routine clinical practice (Fig. 1). The ELBS consortium evolved from the EU IMI project “CANCER-ID” (2015–2019), which aimed to establish standard protocols for the clinical validation of liquid biopsy methods, and pursues the same aims.

As of early 2025, ELBS includes 93 member institutions—55 from academia and 38 from the private sector—spanning 21 countries. This global network also includes members from the North America and Asia. Within the scope of “liquid biopsy,” the ELBS network encompasses the following minimally invasive diagnostic methods: analyses of blood, bone marrow, urine, saliva, stool, cerebrospinal fluid, bronchoalveolar lavage, pleural fluid, along with cells or cell-free components isolated from these materials (e.g., extracellular vesicles, cell-free nucleic acids, platelets). Applications of liquid biopsy include cancer diagnostics, prenatal diagnostics, transplantation medicine, and infection diagnostics, with the current principal focus of the ELBS being in the field of oncology.

The diverse areas of activity within the ELBS consortium are organized into different working groups (WGs): Dissemination/Education, Clinical, Technology, and Regulatory WG. New working groups established at the 2024 GA meeting include “Data Computation” and “Patient Advocate WG”. These working groups are open to all members, their activities are driven by WG leaders and actively shaped by participants. Regular meetings of the individual groups, as well as an annual general assembly meeting, ensure that the ELBS network remains dynamic and interactive. In the following sections, we report on the working groups’ updates presented at the last yearly general meeting, which was held in Barcelona, November 2024.

## Education and dissemination

The Education & Dissemination Work Group (WG) plays a pivotal role in fostering strong networks and meaningful interactions. The group reported on their key activities of a) Welcoming new members, b) Building Connections and partnerships with other scientific Societies, c) Participating to Conferences and Workshops worldwide where ELBS activities are presented; d) Co-Organization of Congresses; e) Raising awareness; f) Fostering Educational Programs.

A central focus of ELBS dissemination efforts is raising awareness about the applications of liquid biopsy, not only in translational research but also in routine clinical practice. Workshops at European and international levels in 2024 were organized to share knowledge across scientific and medical domains. These efforts included In-person and online presentations (see below), collaborations with pharmaceutical and biotechnology companies to ensure the sustainability and integration of liquid biopsy in clinical care.

ELBS is committed to education in the field of liquid biopsy on the basis of the most recent data published [2, 3] through initiatives such as the COST Action program, piloted in Aarhus, Denmark or the European Master of Molecular Pathology (EMMP) at the University of Nice Côte d’Azur, France [4].

ELBS has established partnerships with prominent scientific societies, such as the European Society of Pathology (ESP) and the International Society for Extracellular Vesicles (ISEV). In 2024, ELBS supported notable events such as the 8^th^ Liquid Biopsy Research Meeting in Tokyo (Japan), CNAPS in Graz (Austria) and NORD Workshop in Hamburg (Germany). The WG is actively involved in the organization of the upcoming 14^th^ International Symposium on Minimal Residual Cancer Disease (ISMRC)set for May 7–9, 2025, in Nice, France.

ELBS also publishes original research, review articles, and standardized guidelines for liquid biopsy protocols [5]. Current efforts include developing RECIST criteria for ctDNA and drafting a white paper on integrating liquid biopsy into healthcare systems.

## Technology assessment

The Technology WG consists of 3 subgroups focusing respectively on CTCs, ctDNA, and EVs. Here, we will summarize the reported activities of these 3 subgroups.

### Circulating tumor cells (CTCs)

The circulating tumor cell Technology Working Group (CTC-WG) within ELBS brings together scientists, clinicians, and industry partners to advance CTC research and its clinical applications. In addition to serving as prognostic markers, CTCs offer unique advantages in liquid biopsy, providing genomic insights alongside phenotypic and functional information from intact, viable tumor cells. This capability facilitates the exploration of tumor heterogeneity, metastatic potential, and expressed therapeutic targets, complementing other liquid biopsy biomarkers such as ctDNA. Consequently, CTCs hold promise for accurate, minimally invasive cancer diagnosis, prognosis, treatment monitoring, and response prediction. The group emphasizes collaboration, harmonization, and validation of innovative technologies to maximize the clinical impact of CTCs.

The 2024 meeting in Barcelona marked significant growth for the CTC-WG. At the General Assembly the primary goal of the CTC-WG was reinforced, which is to address key challenges in the field, such as variability in methods, lack of standardization, and the need for clinically validated assays. Short-term goals include creating an inventory of technologies, analytes, and biomarkers used by ELBS member laboratories to inform ring trials, validate novel platforms, and identify funding opportunities. Long-term objectives focus on developing standardized protocols (ISO-15189), external quality control (EQA) schemes, and clinically validated CTC assays that meet the highest standards.

The progress made by the CTC-WG was summarized during the meeting, accompanied by two selected presentations showcasing the advances of ELBS members in the CTC field. 1) “Decoding prostate cancer resistance: Why high-quality samples and standards matter” was presented by Amin El-Heliebi from Medical University of Graz, Austria; and 2) “Longitudinal evaluation of CTC to predict recurrence risk in early HCC treated with local ablative therapy: the INTENT study” was presented by Marianna Alunni-Fabbroni from LMU Klinikum, Germany. A report on the quarterly online meetings was presented. These meetings provide a platform for scientific exchange and for discussions about novel methodologies, recent publications, and collaborative projects. CTC-WG meetings consistently attract 50–70 participants from both academic and industry backgrounds, reflecting a high level of engagement and relevance. A major milestone was the establishment of the CellSearch-EQA ring trial involving nine leading European (Toulouse, France; Hamburg, Germany; Oslo, Norway; Santiago de Compostela, Spain; Athens, Greece; Düsseldorf, Germany; Rotterdam, Netherlands), which assessed the robustness and reproducibility of this widely used technology, laying the groundwork for future ring trials, cross-comparisons and a forthcoming publication. Results were shared and plans for another ring trial of the Parsortix® system from Angle were initiated. Additionally, efforts to technically validate emerging technologies demonstrate the group’s commitment to technological innovation and harmonization.

The CTC-WG also connects technological advancements with clinical needs by collaborating with the ELBS Clinical Working Group. This partnership identifies high-priority clinical questions and develops CTC assays to address these challenges, aligning research with real-world applications.

The goal remains to advance CTC technologies toward clinical implementation, focusing on molecular tumor boards and precision oncology. By fostering collaboration and addressing both technical and clinical challenges, the CTC-WG has become a cornerstone of the ELBS community. Its focus on scientific exchange, technical validation, and clinical integration drives progress in CTC-based liquid biopsy research, contributing to improved cancer outcomes across Europe and beyond [6–10].

### Circulating tumor DNA (ctDNA)

The ctDNA Technology Working Group of the ELBS is dedicated to advancing the clinical application of circulating tumor DNA (ctDNA) as a liquid biopsy tool. Our efforts aim to tackle technical and procedural challenges and to facilitate the reliable use of ctDNA diagnostics in oncology and other medical disciplines [5, 11–13]. Alongside online meetings, participation in international congresses, and engagement in EU projects (e.g., CAN.HEAL), the most comprehensive initiative aligned with our vision was the two-day ‘ctDNA Expert Workshop on Quality Assessment and Reporting’, which took place at the Barcelona Biomedical Research Park (PRBB) on October 5–6, 2023.

**Fig. 2:**
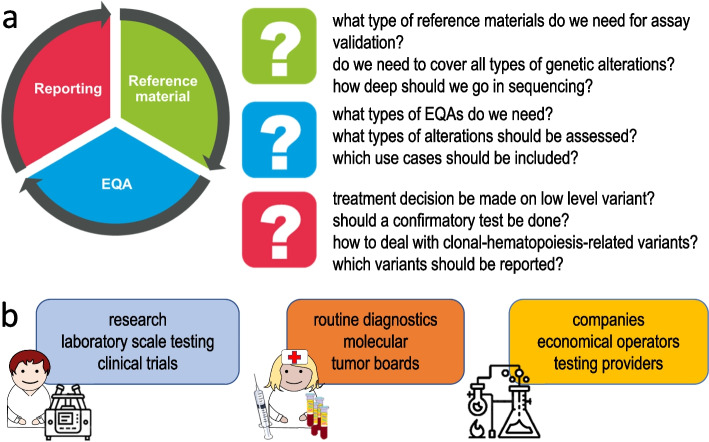
ctDNA WG activities (**a**) the ctDNA Barcelona ELBS workshop: areas and questions. **b** ctDNA: a survey by the European Liquid Biopsy Society (ELBS), topics and potential responders

The General Assembly meeting provided an opportunity to inform the entire ELBS community about the organization and outcome of the Workshop. The structured consensus-building process was described: key controversial topics were identified in advance, a pre-workshop questionnaire was distributed to all the participants, and a state-of-the-art summary with key challenges were presented by both academic and industry representatives. During the workshop, parallel expert discussion groups were convened, interspersed with plenary sessions to facilitate extensive multidisciplinary exchange of ideas. Expert opinions were anonymously collected using real-time online tools during the workshop and supplemented by a post-meeting questionnaire enabling objective measurement of consensus (Fig. 2a).

Three key workshop topics were extensively elaborated during the meeting: (a) the development of reference standards for laboratory quality control, (b) external quality assessment (EQA), and (c) diagnostic reporting. Attention was drawn on the scope and structure of an optimal EQA program, and the prioritization of reference standards for clinically relevant genomic variants across various tumor types and clinical indications. The essential components of diagnostic reports were discussed, including patient clinical features, sample quality, assay specifications, and reporting metrics. Finally, workshop recommendations were described on how to report challenging or controversial variants, address negative results, and manage unexpected or incidental findings. The outcomes of the workshop have been published recently [5].

The ctDNA Working Group also underscored the need to engage a broader ctDNA user community extending beyond ELBS. To this end, a comprehensive ELBS survey was announced. The survey will assess the current state of ctDNA testing for both research and diagnostics across the EU (Fig. 2b). Participation in the survey, online *here*, is not limited to ELBS members and is warmly encouraged from all stakeholders engaged in the implementation of liquid biopsy. In a specific section, respondents may provide their opinions and share their vision for the future of ctDNA testing, ensuring the survey captures innovative ideas and diverse perspectives.

All these initiatives together will allow to gather information relevant to better focus on enhancing patient access to liquid biopsy diagnostics, tackling unique challenges and opportunities throughout Europe and accelerate the regulatory approval and reimbursement processes for ctDNA technologies. In this context, to be mentioned that ELBS collaborates also with the Dutch COIN consortium on implementation of ctDNA testing in clinical practice.

### Extracellular vesicles

Extracellular vesicles (EVs) are micro- or nanoscale structures released by all eukaryotic and prokaryotic cells. Once released, they diffuse into their environment and interact with target cells, performing a wide array of functions. EVs are enclosed by a membrane that mirrors the structure of the originating cell’s plasma membrane, consisting of a lipid bilayer displaying extracellular protein and sugar domains. During their formation, EVs are actively or passively loaded with a variety of molecules, including lipids, proteins, and nucleic acids. Additionally, their membranes are often coated with a diffuse and uneven biomolecular corona, influenced by their surroundings and cellular context, which can impact their destination and function [14–16]. As natural carriers of biomolecules which mirrors cell of origin EVs hold immense potential for clinical applications as they serve as non-invasive liquid biopsy biomarkers and as therapeutic targets [17–20]. The overarching goal of the EV-WG is to accelerate the translation of EVs as diagnostic tools into clinical applications. To achieve this, we 1) connect societies, 2) overcome methodological challenges, and 3) position EVs in the liquid biopsy field.

#### Connecting societies: ISEV-ELBS intersociety working group

The prime activity of the EV working group involved the establishment of a connection with the International Society of Extracellular Vesicles (ISEV). To this end, the ISEV-ELBS Intersociety Working Group was established. Its goal is to foster collaboration between the two societies to promote the reproducibility of liquid biopsy-EV preparation and analysis to encourage discussion on EV research and its applications in liquid biopsy approaches. Together the two societies will co-host meetings, develop collaborative initiatives and report developments in the EV-liquid biopsy field.

#### Overcoming methodological challenges - interdisciplinary collaboration, sharing and education

Over the past two decades, methodological advancements have significantly expanded the tools available for preparing and characterizing EVs, enhancing research and translational applications. However, the wide array of available methods makes it difficult to determine the best suited for a specific experiment. This challenge is further compounded by the heterogeneity of EVs and the complex composition of liquid biopsies [14]. To address these methodological challenges in EV-based diagnostics, the second activity of the EV working group focused on promoting interdisciplinary collaboration (involving industry, scientists, and clinicians), fostering the exchange of knowledge (including standard operating procedures and guidelines), and supporting education. To this end, the EV working group organized an online meeting in June 2024 to share expertise on liquid biopsy collection and processing for downstream EV assessment. This discussion included leaders from the scientific reproducibility task forces of ISEV, focusing on blood, urine, and cerebrospinal fluid. The composition of blood and its derivatives (i.e., plasma and serum) are donor-dependent and is influenced by collection and processing steps [21]. Since there are hundreds of pre-analytical protocols and over forty variables, developing standard operating procedures for EV analysis remains challenging. Consequently, the Blood EV Task Force proposed standardized reporting of (i) the applied blood collection and preparation protocol and (ii) the quality of the prepared plasma and serum [22]. Also, the urinary EV and the CSF task forces developed liquid biopsy-specific recommendations and reporting guidelines [23, 24].

#### Position EVs in the liquid biopsy field

Overall, EV analysis of liquid biopsies requires adequate consideration of pre-analytical variables [25]. To overcome these methodological challenges, the EV working group will continue to strengthen interdisciplinary collaborations and ensure the exchange and distribution of methodological knowledge. EVs are easily accessible in diverse liquid biopsies, offer perspectives for multi-analyte biomarker measurements (including nucleic acids and proteins), are present in high concentrations and are relatively stable [14, 26]. DNA can be bound to proteins (including the nucleosome, as canonically reported), associated to EVs or encapsulated in EVs [27]. Similarly, RNA can be protected from degradation in ribonucleoprotein complexes or EVs [28]. To discuss the complementarity or redundancy of different liquid biopsy targets, the EV working group organized a meeting in November 2024. This discussion included Dr. Irene Casanova-Salas and Dr. Florent Moulière, who recently investigated the dual roles of cell free versus EV-associated nucleic acids as liquid biopsy targets [27, 28]. Overall, increasing data confirm that EVs are enriched with tumor-derived nucleic acids and have the potential to facilitate the early detection of therapy response and resistance, alongside cell-free nucleic acids.

## Clinical studies

The clinical WG reported on the status and upcoming activities. These include uveal melanoma focusing on diagnostic liquid biopsy in patients with pigmented uveal lesions suspected of uveal melanoma and identifying patients with early dissemination and/or a high risk of relapse. In cutaneous melanoma, an ongoing activity centers on identifying high-risk stage IIB-III patients (Lübeck, Kiel, Hamburg, Freiburg). Furthermore, the WG reported on LB-stratified trial designs in melanoma based on previously reported findings [29, 30]. Using ultra-sensitive ctDNA analysis, the design of a proof-of-concept clinical trial addressing ctDNA-guided therapy with anti-PD-1 for stage IB-IIA melanoma after surgical resection (ADJUliquidSelect) was discussed and a timely realization with partners within ELBS, as well as pharmaceutical companies, is anticipated. Moreover, the proof-of-concept study design of ctDNA-guided perioperative ICI therapy for resectable stage IIIB-IV melanoma (NEOliquidSelect) was presented. The adjuvant part includes an mRNA-based individualized neoantigen therapy arm and an observation-only arm. Lastly, a ctDNA-guided follow-up of high-risk uveal melanoma after surgical resection (liquidSurveillance) was presented and a trial design was discussed.

In GIST, the GISG-19 trial, which identifies MRD in resected Miettinen high-risk patients, has been set up in Germany [30]. Moreover, a liquid biopsy guided treatment stratification trial for GIST patients is being planned at the University Medical Center Essen.

The working group organized a workshop on circulating tumor DNA (ctDNA) in neuroendocrine tumors (NE) on February 20, 2024 in Barcelona. Neuroendocrine tumors pose numerous clinical challenges due to their rarity, the limited number of clinical trials conducted, and the lack of available preclinical models, which significantly hinder the development of new therapies. Furthermore, the absence of clearly defined prognostic biomarkers for clinical use complicates the management of these tumors even more, making NE a particularly challenging area in oncology.

The activities of the Clinical WG as mentioned above are tightly connected with those of the ctDNA WG. The future implementation of ctDNA (and other liquid biopsy markers) into the RECIST criteria for assessing therapy responses in advanced solid malignancies is one of the key activities in ELBS. A review of ctDNA criteria to complement RECIST response criteria in solid tumors will be published later this year. Moreover, an international ctDNA RECIST network is established and a first international symposium on ctDNA-RECIST in Aarhus Denmark, is scheduled to take place on March 14–15, 2025.

A survey has been conducted to gather insights on additional liquid biopsy-guided trial activities within the ELBS. Initial proposals for involving of the ELBS Clinical WG in various clinical trials have already been submitted. These include the Gallop Study, FORCE Study, MRD-GUIDE (Lung), MEDOCC-CrEATE, and Liquid Biopsy Stratified Melanoma Trials [31–33].

## Regulatory issues

The clinical applicability and clinical validity of liquid biopsy biomarker applications have been convincingly demonstrated in numerous studies. For instance, ctDNA testing in cancer patients enables better determination of who to treat, how to treat, and when to adjust treatment. As such, liquid biopsy biomarkers have nearly reached the point where their implementation in clinical practice enhances care for cancer patients. However, the regulatory pathway for implementing innovative molecular diagnostics is fragmented and varies significantly worldwide, including within the EU. For example, while Medicare reimburses several ctDNA tests for specific types of cancer in the U.S.A., the adoption of liquid biopsy biomarker applications to enhance care for EU citizens is lagging. Within the EU, ctDNA biomarkers currently face the risk of being implemented in the clinical care workflow across Europe in a non-evidence-based and uncontrolled manner. This situation poses a risk to the health of European citizens who may receive suboptimal diagnostics, as well as to the healthcare systems due to the lack of control over cost developments.

At the General Assembly meeting, the concept was stressed that the primary objective of the ELBS regulatory Working Group is to facilitate the implementation and integration of liquid biopsy assays into routine clinical practice. To gain a better understanding of the current obstacles and challenges in making liquid biopsy biomarkers available, accessible, and affordable for EU citizens, the ELBS hosted a satellite workshop titled “IVDR & Reimbursement - The future of liquid biopsy adoption in Europe”, which took place in Barcelona on November 27, 2024. By gathering insides from numerous experts in the field, the workshop effectively highlighted the diversity and complexity of issues that must be addressed to achieve successful implementation. Key aspects include the technical and clinical performance of tests, the regulatory steps required for IVDR approval, demonstrating the clinical utility of a test, and the regulatory pathways for reimbursement.

One approach to systematically review the necessary steps for implementation is to compare the introduction of a biomarker test in clinical practice with the introduction of a novel drug, for which clear guidelines outline the criteria that should be met and regulatory authorities like the EMA evaluate and approve new medications. Currently, liquid biopsy predictive biomarkers can be assessed according to this process as companion diagnostics. However, a regulatory framework is lacking for biomarkers used in early detection, prognosis/risk assessment, or monitoring treatment response. Several pressing questions remain to be addressed, such as: How should analytical test requirements be defined for a specified intended use in a defined population? What clinical benefits must be demonstrated, and what cost is deemed acceptable? Is it essential to conduct randomized controlled trials for each biomarker application, or can we utilize real-world data as an alternative method for evidence-based introduction of biomarker assays? Additionally, once approved at the EU level, what regulatory mechanisms are in place in various EU member states to provide such a test to citizens?

The excellent and lively discussions emphasized the need for a robust regulatory framework for biomarker implementation, including regulatory authorities dedicated to specifying, assessing, evaluating, and approving biomarkers in a standardized and controlled manner. Collaboration between regulatory agencies, healthcare providers, industry stakeholders, academic institutions, medical professionals, and patient advocacy groups is essential to develop comprehensive guidelines and standards for the clinical implementation of innovative biomarkers.

The workshop prompted several action items for the ELBS regulatory WG, among which:

- Write a position paper to increase knowledge and awareness about the current hurdles and gaps in the biomarker implementation process.
- Install expert review teams, to provide recommendations for guidelines.
- Seek dialogue with and ask advice from the EMA; formulate a best-practice regulatory framework for EU biomarker regulatory acceptance and implementation.
- Seek endorsement by (medical professional) societies (e.g. ESMO and others)
- Seek dialogue with and ask advice from EU member state national regulatory authorities; formulate a best-practice regulatory framework for national biomarker regulatory acceptance and implementation.

## Data computation

Data Computation is currently confronting significant challenges, particularly in genomics analysis, due to data’s overwhelming volume and complexity that strain traditional analytical methods. Simultaneously, AI-based machine learning and deep learning technologies face difficulties with pattern recognition, predictive modeling, and multi-omics/modal data integration, necessitating high-quality data and sophisticated algorithms to synthesize diverse biological information. Computational researchers also grapple with challenges such as real-time survival analysis and comparative studies, which are crucial for interpreting patient outcomes and assessing treatment efficacy. Furthermore, data visualization and clinical decision support systems face challenges regarding effective visualization and interpretation of complex datasets, which is essential for informing healthcare professionals’ decisions. At the meeting, the discussion focused on how these challenges will be critical for advancing research and enhancing precision patient care in the rapidly evolving field of medical science and liquid biopsy.

In response to these issues, the medical and epidemiological research landscape has evolved significantly in recent years, driven by technological advancements and a better understanding of complex biological systems. This evolution necessitates the establishment of clear guidelines and workflows for data handling, harmonization, linkage, and analysis, especially in genomic studies, multi-omics, and data integration, where valuable health insights may be discovered [34, 35].

The Data Computation WG pointed out that organizations like the ELBS are pivotal in this space, offering resources and support for data analytics through facilitating discussions among working groups and gathering input from field experts to develop comprehensive and reproducible workflows. These workflows will be designed to manage all aspects of data handling—from collection and storage to preprocessing and integration—equipping researchers with essential analytical tools.

A key objective of the ELBS initiatives is to foster collaboration among the scientific community. Promoting open sharing of code and analytical methods will enhance reproducibility and reduce duplication of efforts to maximize discovery and innovation. This collaborative spirit enhances research quality and cultivates an inclusive environment conducive to testing and refining diverse methodologies.

Another essential aspect will be the training young scientists and technicians. By offering workshops and practical training sessions, the ELBS aims to equip the next generation of researchers with the skills necessary to handle complex datasets confidently. This initiative meets the dual goals of individual skill development and fostering a broader scientific community capable of addressing future challenges.Continuous learning is vital as the field of data handling rapidly evolves. The ELBS promotes structured training and collaborative initiatives, ensuring that researchers remain informed and actively contribute to the ongoing development of innovative solutions.

ELBS aims to cultivate an informed and skilled community ready to navigate the complexities of medical and epidemiological research through collaboration, expert-led webinars, and training for young scientists. Initiatives such as surveys and upcoming webinars scheduled for 2025 will further strengthen collaborative efforts and knowledge sharing among members, reinforcing ELBS’s commitment to fostering a supportive research environment.

## Closing remarks

The 2024 General Assembly Meeting ELBS represented a great opportunity to bring together experts from academic and clinical research, innovative small and medium-sized enterprises (SMEs), diagnostics companies, and the pharmaceutical industry, to elaborate on the establishment of a unique framework for translating various liquid biopsy applications into clinical practice. The main presentations focused on the development of standardized guidelines, planning and reporting of ring studies for various technologies (CTC, EVs, ctDNA), running clinical studies and training in the field of liquid biopsy for physicians and researchers from related disciplines. The ELBS working groups will further explore the complementarity and redundancy of different liquid biopsy targets and investigate the possibility of maximizing the analysis of different biomarker analytes starting from one blood draw to support comparative assessments. We are thankful to all the participants and invite readers to update themselves about the activities and future events of ELBS at the following link https://elbs.eu.

## Data Availability

No datasets were generated or analysed during the current study.
